# Robust Underwater Docking Visual Guidance and Positioning Method Based on a Cage-Type Dual-Layer Guiding Light Array

**DOI:** 10.3390/s25206333

**Published:** 2025-10-14

**Authors:** Ziyue Wang, Xingqun Zhou, Yi Yang, Zhiqiang Hu, Qingbo Wei, Chuanzhi Fan, Quan Zheng, Zhichao Wang, Zhiyu Liao

**Affiliations:** 1 State Key Laboratory of Robotics and Intelligent Systems, Shenyang Institute of Automation, Chinese Academy of Sciences, Shenyang 110016, China; wangziyue23@mails.ucas.ac.cn (Z.W.); yangyi@sia.cn (Y.Y.); hzq@sia.cn (Z.H.); weiqingbo22@mails.ucas.ac.cn (Q.W.); fanchuanzhi@sia.cn (C.F.); zhengquan@sia.cn (Q.Z.); wangzhichao@sia.cn (Z.W.); liaozhiyu@sia.cn (Z.L.); 2 University of Chinese Academy of Sciences, Beijing 100049, China; 3 School of Electronic and Information Engineering, Harbin Institute of Technology, Shenzhen 150001, China

**Keywords:** AUV recovery, AUV docking, visual positioning, optical guidance, dual-layer light array

## Abstract

Due to the limited and fixed field of view of the onboard camera, the guiding beacons gradually drift out of sight as the AUV approaches the docking station, resulting in unreliable positioning and intermittent data. This paper proposes an underwater autonomous docking visual localization method based on a cage-type dual-layer guiding light array. To address the gradual loss of beacon visibility during AUV approach, a rationally designed localization scheme employing a cage-type, dual-layer guiding light array is presented. A dual-layer light array localization algorithm is introduced to accommodate varying beacon appearances at different docking stages by dynamically distinguishing between front and rear guiding light arrays. Following layer-wise separation of guiding lights, a robust tag-matching framework is constructed for each layer. Particle swarm optimization (PSO) is employed for high-precision initial tag matching, and a filtering strategy based on distance and angular ratio consistency eliminates unreliable matches. Under extreme conditions with three missing lights or two spurious beacons, the method achieves 90.3% and 99.6% matching success rates, respectively. After applying filtering strategy, error correction using backtracking extended Kalman filter (BTEKF) brings matching success rate to 99.9%. Simulations and underwater experiments demonstrate stable and robust tag matching across all docking phases, with average detection time of 0.112 s, even when handling dual-layer arrays. The proposed method achieves continuous visual guidance-based docking for autonomous AUV recovery.

## 1. Introduction

Autonomous Underwater Vehicles (AUVs), capable of autonomous navigation and operation, have been extensively utilized in fields such as marine resource exploration, scientific research, seabed mapping, and military target detection, demonstrating irreplaceable value particularly in hazardous or inaccessible underwater environments [1,2]. Autonomous operation of AUVs significantly reduces human intervention, thereby enhancing operational safety and efficiency.

However, limited internal space and energy storage capacity necessitate periodic energy replenishment, data transmission, and routine maintenance for AUVs, placing high demands on the efficiency and reliability of docking and recovery technologies. Existing recovery guidance approaches are mainly categorized into capture docking, platform docking, and tapered port docking methods [3]. Among these methods, tapered port docking is the most widely adopted due to its simplicity and high docking success rate, typically employing funnel-shaped [4] or box-shaped [5] axial docking structures.

Based on different sensing modalities, the underwater recovery positioning methods for AUVs are classified primarily into acoustic [6,7], electromagnetic [8,9] and visual [10,11] approaches. Acoustic and electromagnetic methods are typically used for coarse, long-distance positioning and are predominantly employed during the homing stage. Precise docking requires AUVs to approach the docking station with controlled velocity and high accuracy. Consequently, visual positioning methods utilizing optical sensors have gained prominence owing to their superior accuracy and rapid response features. Beyond docking, optical technologies have also been widely studied for underwater wireless communication and energy supply [12,13,14,15], demonstrating their versatility as enabling.

For precise positioning during visual docking, AUVs utilize onboard cameras to detect predefined visual markers. These markers typically fall into two categories: active beacons and passive markers. Active beacons are predominantly composed of lights. For instance, Li et al. [16] developed a wide-area single-beacon visual guidance system, which, by analyzing the morphological changes and movement trajectories of a single light spot, offered a concise and practical localization solution for low-cost and lightweight AUVs. However, a single light beacon usually provides guidance only for the dominant direction, thereby limiting its overall utility. Consequently, multi-beacon localization approaches are generally preferred. Nevertheless, multi-beacon scenarios frequently present challenges such as occluded light sources and spurious detections, complicating accurate matching. To address these issues, Yan et al. [17] proposed a four-degree-of-freedom visual positioning algorithm that employs an L-shaped light array installed beneath a docking structure. Their method leverages the geometric configuration of the light array to robustly identify valid beacons even under partial occlusion. Similarly, Xu et al. [18] utilized four symmetrically arranged green LEDs on a docking ring for stereo camera-based localization, effectively eliminating spurious light sources by analyzing their specific topological structure. The second category comprises passive visual markers, utilized primarily for short-range precise positioning. Ren et al. [19] introduced ArUco markers combined with blue-green light positioning to facilitate precise close-range guidance. Zhao et al. [20] enhanced the effective positioning range of AUVs with limited visual fields by deploying multiple ArUco markers. Wei et al. [21] proposed an enhanced AR-coded visual marker system combined with an image restoration model tailored for underwater environments, significantly improving visual marker detection robustness and localization accuracy.

Existing visual positioning methods guided by beacons or markers continue to exhibit notable limitations. A single-layer planar light array often causes key localization beacons to fall outside the camera’s limited field of view during AUV docking, resulting in discontinuous and unreliable localization. While smaller visual markers can offer improved positioning precision and enhanced adaptability to field-of-view variations, their passive nature significantly restricts detectability over longer distances or in highly turbid underwater environments.

The main contributions of this paper are as follows:

(1) A visual guidance scheme based on a dual-layer light array is proposed to mitigate the limitations of onboard visual systems with restricted fields of view. By optimizing the spatial configuration of the light sources, the scheme ensures continuous target visibility within the docking station and significantly enhances the reliability of the autonomous docking process.
(2) Based on this guidance scheme, a corresponding visual localization method for the dual-layer guiding light array is presented. This method dynamically distinguishes between the front-layer and rear-layer light sources at each docking stage, ensuring stable optical guidance with at least one layer at any given time. When the number of detected light sources in a single layer reaches four or more, the method performs robust tag matching and achieves pose estimation in world coordinates. The localization of each layer is performed independently to guarantee the robustness of the overall system.
(3) To verify the effectiveness and accuracy of the proposed method, a series of simulation and pool experiments were conducted. The experimental results demonstrate that the proposed method not only adapts effectively to changes in the field of view but also robustly addresses issues such as missing and spurious light beacons, thus significantly improving the robustness of the AUV autonomous docking process.

The remainder of this paper is organized as follows. Section 2 introduces the dual-layer cage-type guide light array position scheme, including the design rationale and phase-wise analysis of the docking process. Section 3 presents the visual positioning method based on the dual-layer light array, covering light extraction and discrimination, the robust tag-matching framework with PSO optimization and backtracking EKF, and pose estimation techniques. Section 4 describes the comprehensive experimental validation, including simulation studies of the tag matching framework and pool-based feasibility experiments for continuous guidance. Finally, Section 5 concludes the paper with a summary of key contributions, quantitative results, and discussion of future work directions.

## 2. Dual-Layer Cage-Type Guide Light Array Position Scheme

This section introduces a cage-type docking scheme for AUV recovery based on a dual-layer guiding light array. By strategically arranging light sources both inside and outside the docking station, the system ensures continuous visual localization throughout all docking phases, from initial target search to final docking.

### 2.1. Design of Dual-Layer Light Array for Cage-Type Docking System

Given the substantial number of LEDs employed in both the front-layer and rear-layer arrays, the configuration must enable the downstream visual algorithm to accurately distinguish individual lights tags. To achieve this, the light array design must satisfy the following criteria:

(1) Front-layer light array: The front layer should support long-distance detection and adopt a dispersed, asymmetrical configuration to mitigate light overlap or merging caused by optical diffusion.
(2) Rear-layer light array: The rear layer should adopt a compact layout with lights featuring narrow beam angles, making it suitable for operation in restricted field-of-view scenarios and ensuring reliable detection at short distances.
(3) Structural features: The overall arrangement should present distinct spatial patterns, allowing the AUV to accurately differentiate and match individual light beacons as they progressively enter the camera’s field of view.
(4) Deployment location: Since AUVs typically approach the docking station while ascending from deeper to shallower depths, the front-layer light array should be primarily deployed near the lower section of the docking station to facilitate early detection and localization.

**Figure 1:**
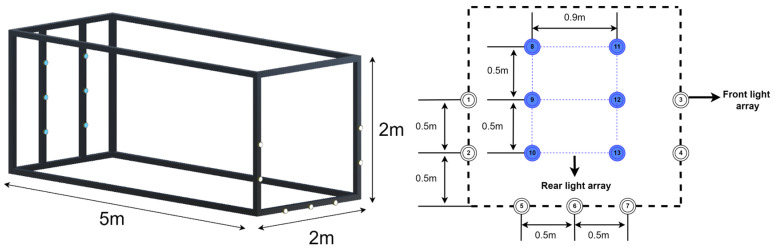
Layout of the cage-type recovery system with dual-layer light arrays.

Based on the aforementioned requirements, a dual-layer light source configuration was designed, as illustrated in Figure 1. The dimension of the docking station is 2 × 2 × 5 m. In this configuration, the front-layer light array consists of seven high-intensity light sources arranged along the left and right sides and the lower part of the entrance of the docking station. To enhance distinguishability, white light sources are used in the front layer, while blue light sources are employed in the rear layer. The numbering of the light sources is shown in the front-view schematic (right side of Figure 1).

The color selection is based on both detection performance and underwater optical properties. White LEDs are employed for the front layer because their broadband spectrum (400–700 nm) produces strong camera responses across the visible spectrum, enabling reliable spot detection and segmentation at long ranges where signal strength is critical. Blue LEDs are used for the rear layer, leveraging the fact that blue wavelengths (455–460 nm) experience the least attenuation in underwater environments, thus providing more stable and consistent signals at medium-to-short detection ranges. The distinct spectral difference between white and blue also facilitates reliable layer discrimination during the detection and matching process, reducing the likelihood of false associations between front and rear layer beacons.

### 2.2. Phase-Wise Analysis of Docking Process

**Figure 2:**
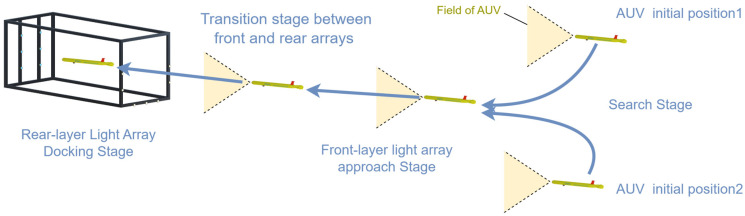
AUV cage-type docking scenario.

**Figure 3:**
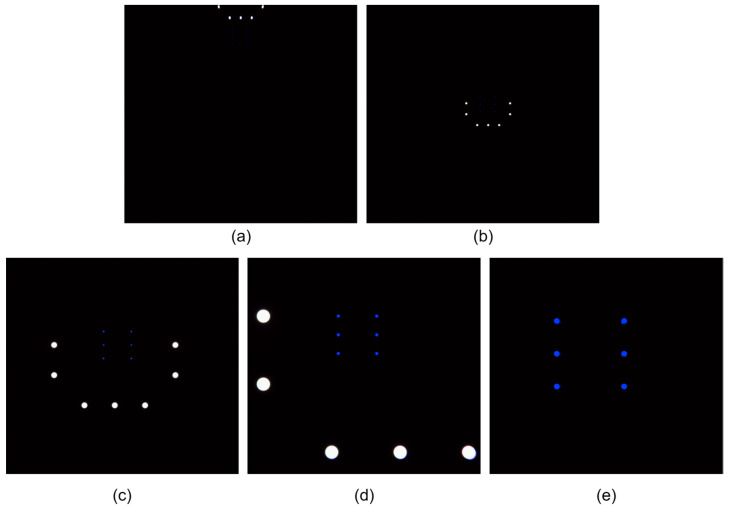
Camera views during the four-stage docking process. (**a**) Search stage: partial front-layer lights visible at distance; (**b**) Front-layer approach stage: all front-layer lights fully visible in field of view; (**c**) Transition stage (early phase): rear-layer lights beginning to appear alongside front-layer lights; (**d**) Transition stage (late phase): some front-layer lights moving out of view while rear-layer lights become dominant; (**e**) Rear-layer docking stage: front-layer lights completely out of view, only rear-layer lights visible for final alignment.

Figure 2 illustrates the proposed cage-type docking scenario. An AUV equipped with a forward-looking camera detects the guiding-light array on the docking station and autonomously executes navigation and docking. To maintain seamless, robust pose estimation throughout the maneuver, the process is organized into four consecutive stages, with representative camera views shown in Figure 3.

1. Search stage: The AUV approaches the docking station from a distance and performs small-scale vertical and lateral maneuvers to search for the front-layer light array. During this phase, the system estimates the position of the docking station based on partially detected front-layer lights. Due to factors such as light attenuation and relative positioning, typically only a subset of the front-layer lights is visible, while the rear-layer lights are out of view (Figure 3a).
2. Front-layer light array approach stage: As the AUV moves closer, the front-layer lights fully enter the camera’s field of view and can be reliably detected. The system utilizes these lights for precise localization and navigation. At this point, the rear-layer lights begin to gradually appear (Figure 3b).
3. Transition stage between front and rear arrays: As the AUV continues to advance, the rear-layer lights progressively enter the field of view. The system must dynamically distinguish between rear and front layer light sources to ensure a smooth transition from front-layer-array-based guidance to rear-layer-array-based guidance, avoiding tracking errors or interruptions (Figure 3c,d).
4. Rear-layer light array docking stage: Once the AUV enters the interior of the docking station, only the rear light array remains visible. At this stage, the system relies entirely on the rear light array for fine-grained localization and attitude adjustment, ensuring accurate and stable final docking (Figure 3e).

## 3. Visual Positioning Method Based on Dual-Layer Light Array

For continuous-guidance localization using a dual-layer guiding light array, we propose the following method. We begin by detecting light sources with conventional vision techniques and then dynamically classify them into front and rear layers based on color or size. Once the layers are separated, our algorithm addresses multi-source matching challenges by applying a robust matching framework that guarantees stable light beacons tag matching under complex conditions, thus enabling robust pose estimation via the sequential quadratic programming-based perspective-n-point (SQPNP) algorithm.

### 3.1. Extraction and Discrimination of Front and Rear Layer Light Arrays

#### 3.1.1. Light Source Feature Extraction

To extract the target light sources from the image, a series of preprocessing steps are performed. First, the color image is converted to grayscale, the binarization threshold is automatically determined using the interval-adaptive OTSU method [22]. Canny edge detection is then employed to extract contours from the binarized image, followed by the identification of connected regions.

To address edge overlap caused by light source halo diffusion, a distance transform is introduced, and its output is used to define a threshold for morphological opening, which refines the connected domains. Subsequently, least-squares circle fitting is applied to the boundary points of each connected region to calculate shape descriptors such as roundness and compactness. These metrics are used to filter out spurious light sources caused by noise or shapes that do not meet geometric constraints, effectively isolating valid light sources for subsequent matching.

#### 3.1.2. Discrimination of Front and Rear Layer Light Arrays

Given that the dual-layer light array becomes sequentially visible under varying observation conditions, the system first differentiates the front and rear layers to maintain robust tag matching.

After detecting the image region corresponding to the light source, the RGB image is converted to the HSV color space. Using predefined HSV thresholds, light sources are preliminarily classified into two categories: blue-channel (rear-layer) and non-blue-channel (front-layer).

In addition, a radius-based decision method is proposed to adaptively classify light sources into front- and rear-layer groups. The process proceeds as follows:

The detected light sources are initially divided into two clusters: smaller-radius blue lights representing the rear layer, and larger-radius non-blue lights representing the front layer. The clustering objective is to minimize the total within-cluster variance, formulated as:

(1) $$\underset{C_{1},C_{2}}{min}J(C_{1},C_{2})=\underset{C_{1},C_{2}}{min}(\sum_{x_{j}\in C_{1}}{\left|x_{j}-u_{1}\right|}^{2}+\sum_{x_{j}\in C_{2}}{\left|x_{j}-u_{2}\right|}^{2})$$

Let $C_{1}$ and $C_{2}$ denote the two clusters corresponding to smaller and larger light radii, respectively, with mean values $u_{1}$ and $u_{2}$. Each $x_{j}$ represents a light source. Clustering is performed by minimizing the above objective function.

If the average radius difference between the two clusters exceeds a predefined threshold (i.e., $||u_{2}-u_{1}||>threshold$), the radii of the two clusters are considered a candidate pair for two-cluster separation. To mitigate misclassification caused by spurious light blobs with abnormal sizes, which may interfere with radius-based cluster discrimination, an additional constraint is imposed. Specifically, each cluster must contain at least four light sources. A frame is considered a valid dual-cluster configuration, and the dual-cluster counter m is incremented only if both conditions are satisfied.

**Figure 4:**
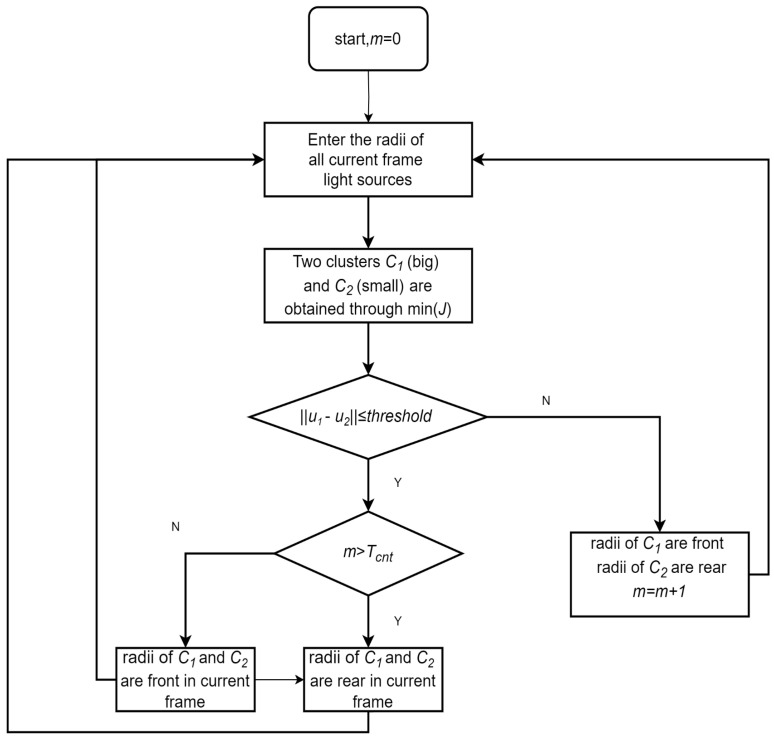
Flowchart for detecting the size of front and rear light arrays.

If the mean radius difference is below the predefined threshold (i.e., $||u_{2}-u_{1}||<threshold$) or if either cluster contains fewer than four light sources, the current frame is considered temporarily unclassifiable. To avoid prematurely discarding valid light sources and to ensure that at least one group of lights is extracted, all detected lights are provisionally treated as belonging to a single cluster. To determine whether this single group corresponds to the front or rear layer, the system refers to the accumulated number of prior frames identified as dual-cluster. If this count m exceeds a predefined threshold *T_cnt_*, the current group is classified as rear-layer light array; otherwise, it is considered to belong to the front-layer light array. Figure 4 illustrates the size detection process.

**Figure 5:**
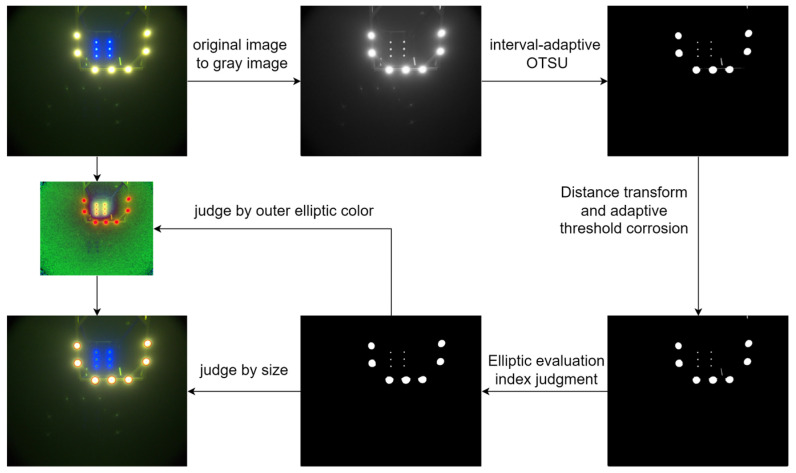
Procedure for extracting and classifying front and rear light arrays.

The complete workflow for light source extraction and discrimination is illustrated in Figure 5.

### 3.2. Single-Layer Light Array Tag Matching Framework

After distinguishing the dual-layer light array, it remains necessary to match the tags within each individual layer. During the AUV’s search phase, the onboard camera may observe the docking station from varying distances and orientations, often resulting in partial detection of the light sources. In the transition phase, as the AUV advances, front-layer lights may gradually exit the field of view, further contributing to incomplete observations. Moreover, failures in distinguishing between front- and rear-layer lights can occur for two main reasons. First, color discrimination may fail because our algorithm classifies a light as “blue” based on the proportion of surrounding pixels falling within a blue hue range. In practice, this threshold may not always be satisfied due to underwater scattering or intensity attenuation. In addition, the diffusion of blue light can overlap with adjacent front-layer white lights, leading to their misclassification as blue. Second, size-based discrimination may fail when the number of detected lights in one layer is insufficient. In such cases, the algorithm may erroneously merge all detected lights into a single class, thereby assigning the entire front layer to the rear layer (or vice versa).

To address light source detection errors—specifically, missing sources caused by partial visibility and spurious detections caused by tag misclassification—this paper proposes a robust tag matching framework that integrates Particle Swarm Optimization (PSO), geometric tolerance filtering, and a backtracking iterative Extended Kalman Filter (BTEKF). First, the initial correspondence between detected beacons and their expected positions is established by optimizing affine transformation parameters under known geometric constraints of the light array, using an enhanced PSO tailored for AUV navigation. To eliminate false matches, a geometric consistency check based on joint distance and angle tolerances is applied, retaining only correspondences that satisfy both thresholds. Finally, when this check identifies erroneous matches in the current frame, a backtracking EKF is triggered: current matches are discarded, a Gaussian motion model is constructed from previous pose estimates, and predicted light source reprojections are used to infer and correct tag assignments in the next frame.

The complete algorithm steps are as follows:

#### 3.2.1. Enhanced PSO for AUV Active Beacon Matching

During the autonomous docking process of the AUV, the geometric relationship between the known beacon positions in the world frame and their detected projections in the image plane can be approximated by a 2D affine transformation. To achieve both efficiency and robustness in beacon matching, we develop an enhanced Particle Swarm Optimization (PSO) algorithm tailored for AUV navigation. A swarm of *N* particles explores a four-dimensional search space, with each particle encoding the transformation parameters: rotation angle *θ*, translations $t_{x}$, $t_{y}$, and scaling factor *s*.

We first normalize the 3D world beacon coordinates to 2D coordinates $P_{w}=\left\{S_{i},i=1,\ldots ,n_{1}\right\}$, by projecting them onto the frontal (x-o-y) plane, discarding the depth component, and applying scale normalization. The image-plane pixel coordinates are normalized as $P_{c}=\left\{R_{j},j=1,\ldots ,n_{2}\right\}$. The goal is to find the affine transform that best aligns these two sets of points. The fitness function is defined as:

(2) $$\underset{i,j}{Fitness(p)=\sum \left||T(\theta ,s)\cdot P_{c}+t-P_{w}|\right|}$$

where the affine transform matrix is:

$$T(\theta ,s)=\left[\begin{array}{cc} s\cdot \cos(\theta ) & -s\cdot \sin(\theta )\\ s\cdot \sin(\theta ) & s\cdot \cos(\theta ) \end{array}\right]$$

Each particle evaluates this fitness, and both its personal best position $p_{best}$ and the global best $g_{best}$ are recorded. Particle velocities and positions are updated by:

(3) $$v_{i}^{t+1}=wv_{i}^{t}+c_{1}r_{1}(p_{best}-x_{i})+c_{2}r_{2}(g_{best}-x_{i})$$

(4) $$x_{i}^{t+1}=x_{i}^{t}+kv_{i}^{t+1}$$

where w denotes the inertia weight; $c_{1}$, $c_{2}$ represent the cognitive and social coefficients, respectively; $r_{1}$ and $r_{2}$ ∈ (0, 1) are uniformly distributed random numbers; and k is a scaling factor for the velocity.

Premature convergence is a common challenge in Particle Swarm Optimization (PSO) [23,24]. To effectively address this and enhance performance for our AUV active beacon matching scenario, we introduce following improvements:

First, refine the learning rate by making it decrease linearly as the number of iterations increases.

(5) $$c_{1}=c_{1BASE}+\left(c_{1FINAL}-c_{1BASE}\right)\left(1-\frac{iter\_count}{MAX\_ITER}\right)$$

(6) $$c_{2}=c_{2BASE}+\left(c_{2FINAL}-c_{2BASE}\right)\left(1-\frac{iter\_count}{MAX\_ITER}\right)$$

$c_{1BASE}$, $c_{1FINAL}$ denote the initial and final values of $c_{1}$, $c_{1BASE}>c_{1FINAL}$, with $c_{2BASE}$, $c_{2FINAL}$ defined similarly. iter_count and MAX_ITER are the current and maximum iteration counts. This linear decay allows faster updates in early iterations and finer adjustments near the end.

Second, since the initial distribution of particles in the search space can affect their update directions, and considering the actual motion range of the AUV docking task, particle parameters of the search space are constrained:

$$\theta \in \left(-10^{\circ },10^{\circ }\right),s\in \left(0.5,8\right),t_{x}\in \left(-1,1\right),t_{y}\in (-1,1)$$

These constraint selections are based on the following physical considerations:

First, for the translation parameters $t_{x}$ and $t_{y}$, we normalize all detected light points by subtracting their mean and scaling them into the [−1, 1] space. This normalization makes the translation estimation dimensionless and robust to scale variations, which justifies our choice of $t_{x}$, $t_{y}$ ∈ [−1, 1].

Second, regarding the roll angle θ, the AUV’s roll motion is physically constrained during docking and cannot be very large. We therefore limit the search range to [−10°, 10°], which covers all realistic roll variations while reducing unnecessary search space in the PSO algorithm.

Finally, the scale factor s is more complex. In an ideal case without spurious detections or missing points, s should be close to 1.0. However, when spurious lights (e.g., distant reflections) are present, they are included in the normalization range, artificially inflating the apparent observation span. For instance, in an extreme case where the docking station is at the bottom of the image and a reflection appears at the top, with the camera at 18.64 m distance and a 60° field of view, the vertical observation span would be approximately 21.52 m. Given that the docking station is only 2 m × 2 m, the theoretical normalized scale could become about 10 times larger. To maintain robustness under such rare but possible cases, we cap the upper bound of s to about 8.

Another extreme case is partial visibility due to missing detections. When the missing points correspond to exactly the upper (or lower) half or left (or right) half of the lights, the effective observation space is halved. This causes the normalization to relatively enlarge the visible points by a factor of two. To compensate, we set the lower bound of s to 0.5, ensuring reliable matching under such extreme scenarios.

Moreover, to ensure continuity in attitude changes during updates, an adaptive penalty function *Penalty*′ is introduced when the updated angle deviates excessively from the previous frame:

(7) $$Penalty^{\prime }=\left\{\begin{array}{cc} 0 & \left|\theta_{now}-\theta_{used}\right|<10{}^{\circ}\\ Penalty*\left(1+\frac{\left|\theta_{now}-\theta_{used}\right|-10}{5}\right) & \mathrm{else} \end{array}\right.$$

Furthermore, we define a composite fitness function that simultaneously accounts for the registration accuracy in the current frame and the pose-continuity with the previous frame:

(8) $$Fitness\left(p\right)=\sum w_{1}\left|T\left(\theta ,s\right)\cdot P_{C}+t-P_{w}\right|\;+\sum w_{2}\left|T\left(\theta ,s\right)\cdot P_{C}+t-T\left(\theta_{\mathrm{pre}},s_{\mathrm{pre}}\right)\cdot P_{C_{\mathrm{pre}}}+t_{pre}\right|+Penalty^{\prime }$$

$w_{1}$ balances the influence of current-frame registration error, while $w_{2}$ penalizes discontinuities (rotation, translation, scaling) between successive frames.

Finally, to enhance global search capability and avoid particles stagnating in local optima, an intelligent restart mechanism is employed:

Particles whose fitness shows no significant improvement over several iterations are reinitialized randomly before the next update; Restarted particles are redistributed into under-explored regions, increasing swarm diversity and the chance of escaping local traps.

**Figure 6:**
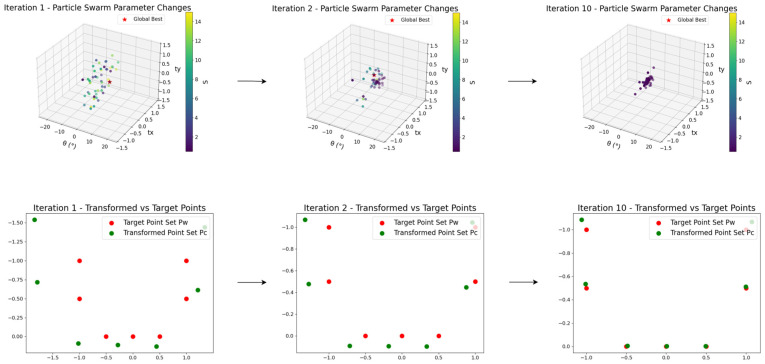
PSO point set registration with tag matching and particle distribution evolution.

Figure 6 illustrates the evolution of the swarm over iterations: the 3D scatter plots show the changes in translation components $t_{x}$, $t_{y}$, and rotation *θ*, while the color gradient encodes the scale parameter *s*.

#### 3.2.2. Consistency of Distance and Angular Ratios

Due to the limited number of PSO iterations and frequent omission of multiple light sources, the raw PSO output may fail to provide accurate tag correspondences.

Let $P=\left\{P_{1},P_{2},\ldots ,P_{n}\right\}$ represent the known beacon coordinates in the world coordinate system (after removing depth), and $p=\left\{p_{1},p_{2},\ldots ,p_{n}\right\}$ denote the corresponding matched points in the image plane obtained by PSO. To robustly validate each candidate correspondence set, we introduce two geometric tolerance criteria:

a. Distance-Ratio Consistency

For every ordered triple (i,j,k), compute the pairwise distance ratios before and after matching:

(9) $$\lambda_{ijk}=\frac{d_{jk}}{d_{ij}}=\frac{\left|P_{j}-P_{k}\right|}{\left|P_{i}-P_{j}\right|},\lambda \prime_{ijk}=\frac{d\prime_{jk}}{d\prime_{ij}}=\frac{\left|p_{j}-p_{k}\right|}{\left|p_{i}-p_{j}\right|}$$

$$\forall i,j,k\;s.t.|1-\frac{\lambda_{ijk}}{\lambda \prime_{ijk}}|<\in_{\lambda }$$

where $\in_{\lambda }$ denotes the maximum allowable relative deviation in the distance ratio.

b. Angular Consistency

For the same triple (i,j,k), compute the interior angle at point $P_{i}$ before and after matching:

(10) $$\theta_{ijk}=\arccos(\frac{\overset{\to }{P_{i}P_{j}}\cdot \overset{\to }{P_{j}P_{k}}}{\left|\overset{\to }{P_{i}P_{j}}\right|\cdot \left|\overset{\to }{P_{j}P_{k}}\right|}),\theta \prime_{ijk}=\arccos(\frac{\overset{\to }{p_{i}p_{j}}\cdot \overset{\to }{p_{j}p_{k}}}{\left|\overset{\to }{p_{i}p_{j}}\right|\cdot \left|\overset{\to }{p_{j}p_{k}}\right|})\;\forall i,j,k\;s.t.|\theta \prime_{ijk}-\theta_{ijk}|<\in_{\theta }$$

where $\in_{\theta }$ denotes the maximum allowable angular deviation.

A candidate correspondence set is accepted only when both geometric criteria are satisfied across all point triplets. Otherwise, a backtracking iterative Extended Kalman Filter (BTEKF) procedure is triggered to re-estimate the tag matching based on historical motion information.

#### 3.2.3. Backtracking EKF Matching Correction

To robustly re-associate beacons and update the AUV’s pose in frames with erroneous tag matches, the predicted pose and its covariance are incorporated into an Extended Kalman Filter (EKF) [25]. The EKF operates in the image space through an iterative predict–correct process to refine the estimated associations.

a. Predictive Covariance Projection

The prior pose $P_{g}$ and its covariance $\sum_{g}^{p}$ are modeled as a single Gaussian distribution. For each known 3D beacon $x_{i}$, the Jacobian $J(x_{i})$ of its 2D projection with respect to the pose is calculated. The aggregate observation-space covariance is then given by:

(11) $$\sum_{i}^{v}=J(x_{i})\sum_{g}^{p}{\left(J(x_{i})\right)}^{T}$$

where $\sum_{i}^{v}$ is the projected covariance of beacon xi in image space, and $J(x_{i})$ represents the partial derivatives of the projection function with respect to pose parameter.

b. Candidate Gating via Mahalanobis Distance

For each beacon $x_{i}$, denote its predicted image projection as $v_{i}$, and let {$u_{j}$} represent the set of candidate points. A measurement $u_{j}$ is accepted only if its Mahalanobis distance to $v_{i}$ satisfies:

(12) $${\left(v_{i}-u_{j}\right)}^{T}{\left(\sum_{i}^{v}\right)}^{-1}(v_{i}-u_{j})\leq M^{2}$$

where M is the chi-square threshold corresponding to the desired confidence level.

c. EKF Update

Process the surviving $\left(x_{i},u_{j}\right)$ pairs in order of increasing gating residual. For each pair, the prior pose and covariance are then updated as follows:

(13) $$K=\sum_{g}^{p}J{\left(x_{i}\right)}^{T}{\left(J(x_{i})\sum_{g}^{p}J{\left(x_{i}\right)}^{T}+R\right)}^{-1}\;p_{g}^{+}=p_{g}+K\left(u_{j}-\mathrm{Proj}\left(p_{g};x_{i}\right)\right)\;\sum_{g}^{p+}=\left(I-KJ\left(x_{i}\right)\right)\sum_{g}^{p}$$

where *K* is the Kalman gain matrix, *R* is the measurement noise covariance matrix, *I* is the identity matrix,$\;\mathrm{Proj}\left(p_{g};x_{i}\right)$ represents the projection of 3D beacon xi onto the image plane using pose $P_{g}$, and the superscript “+” denotes the updated (posterior) estimates.

d. Backtracking Logic

After each update, compute the reprojection residual. If it falls below the acceptance threshold, lock in the match and proceed to the next beacon. Otherwise, discard that candidate $u_{j}$ and try the next. If all candidates for $x_{i}$ fail, backtrack to the previous beacon $x_{i-1}$, re-evaluate its matches, and then resume the forward process.

e. Convergence and Pose Acceptance.

Once the filter has converged (i.e., at least three beacons have been successfully updated), the final pose is recorded. Subsequently, all 3D beacons are reprojected onto the image plane, and among those within a specified pixel–distance threshold, the nearest valid 2D beacon is selected as the initialization reference for SQPNP pose estimation in the next frame.

**Figure 7:**
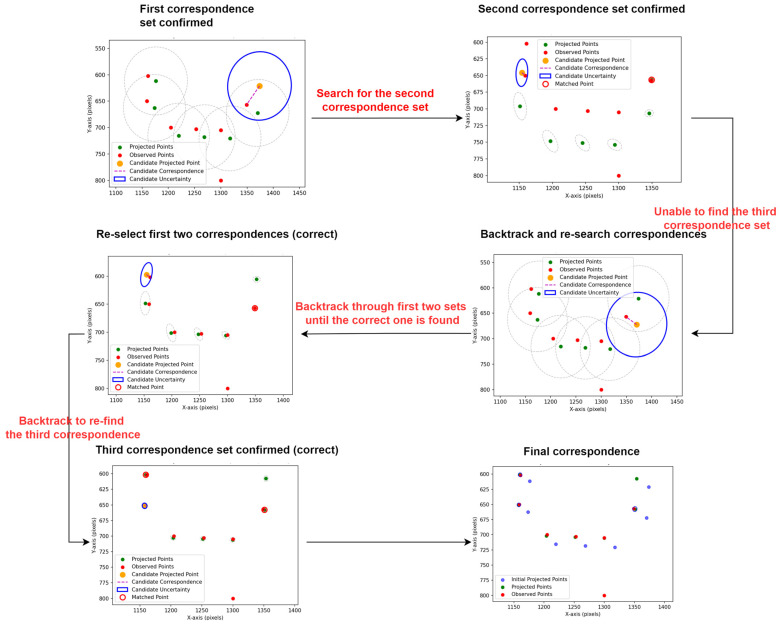
Process Diagram of point set optimization matching via backtracking iterative Kalman filter.

Figure 7 illustrates the complete workflow of the backtracking EKF-driven pose refinement, highlighting a scenario in which one beacon is missing and a noisy point is present.

### 3.3. Pose Estimation

**Figure 8:**
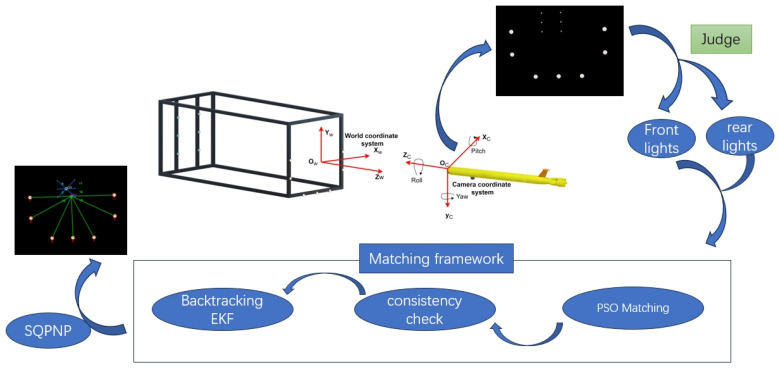
AUV continuous navigation processing framework.

To achieve high-precision pose estimation during the AUV docking process, it is essential to establish a transformation between the world coordinate system and the camera coordinate system (Figure 8). In this work, the Sequential Quadratic Programming for Perspective-n-Point (SQPnP) algorithm [26] is employed to solve the nonlinear PnP problem. This algorithm offers both global optimality and computational efficiency in multi-point matching scenarios, making it well-suited for the pose estimation task involving multiple light source correspondences in the proposed system. In this experiment, SQPNP was used for the front layer light array and the rear layer light array respectively. The complete continuous navigation and localization method described in Section 3 is shown in Figure 8.

## 4. Experiment

To validate the effectiveness and robustness of the proposed dual-layer light array visual localization method, we designed and conducted a series of simulation trials and pool-based experiments, systematically evaluating the algorithm’s performance across a range of representative scenarios.

### 4.1. Single-Layer Light Array Tag Matching Framework

(1) Simulation Environment and Parameter Settings

We built a 1:1-scale simulation environment, an AUV moves toward the docking station along the *Z*-axis at approximately 1.5 m/s, capturing four images per second. Simultaneously, it performs small-amplitude perturbations in the X- and Y-directions (up to ±1 m) and maintains roll, pitch, and yaw angles within ±3°.

(2) Robustness to Missing and Spurious Beacons

To assess the algorithm’s robustness under conditions of partial beacons loss and spurious beacons, we focus on the front-layer light array and design two test scenarios:

a. Randomly remove 1 to 3 front-layer light beacons from the detection results and match using the remaining ones, to evaluate the algorithm’s performance in matching the remaining beacons under conditions of missing beacons.

b. Introduce 1 to 2 randomly moving spurious light beacons into the front-layer light array and test the algorithm’s capability to extract the real beacons from data containing spurious beacons.

**Figure 9:**
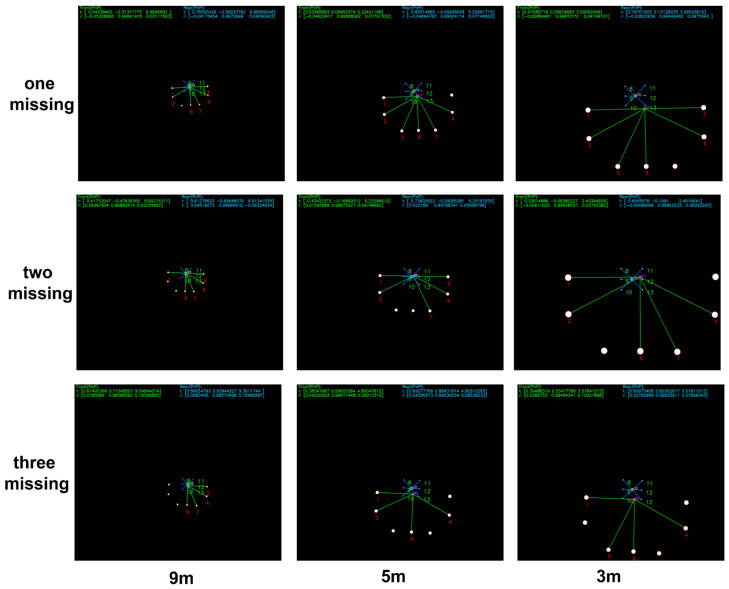
Simulation results under various beacon-loss conditions.

Figure 9 presents the matching and pose estimation results under simulated partial beacons loss, where one, two, or three beacons from the detected set are randomly omitted in each frame to emulate missing observations. The results are shown at different distances of 9 m, 5 m, and 3 m, respectively.

**Figure 10:**
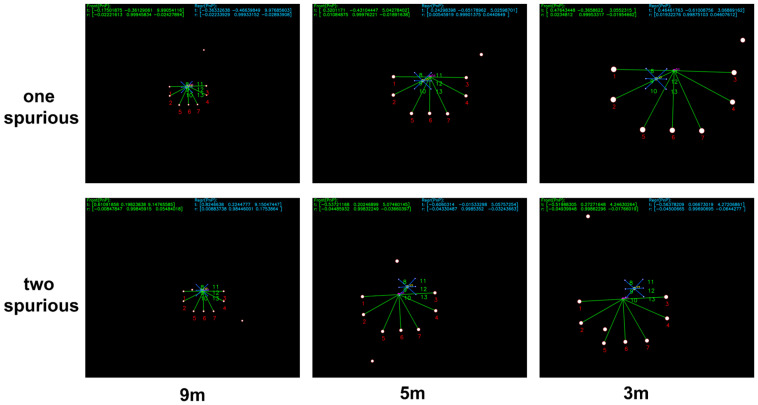
Simulation results in the presence of spurious beacons.

Figure 10 shows the matching results in the presence of one or two injected spurious beacons at distances of 9 m, 5 m, and 3 m. These spurious beacons are randomly added to the detected light set to evaluate the algorithm’s robustness to outliers.

A total of 30 physical-camera trials were conducted in simulation under three conditions: no spurious beacon, one spurious beacon, and two spurious beacons. Across these trials, the front-layer light array was fully detected 2108 times in the absence of spurious lights, 2076 times with one spurious light, and 2153 times with two spurious lights. Additionally, extreme beacon-loss scenarios were evaluated to measure the success rate of matching and pose estimation. The summarized results are presented in Table 1. These results demonstrate that even under the extreme condition of three missing beacons, the PSO-based matcher alone achieves a 90.3% correct matching rate, highlighting its strong initialization capability. During the search phase, the algorithm maintains high success rates despite the absence of some beacons. In the subsequent guided phase, the integration of PSO with the backtracking EKF achieves a perfect 100% matching rate. Moreover, the PSO algorithm effectively suppresses spurious beacons and accurately identifies the real beacons.

**Figure 11:**
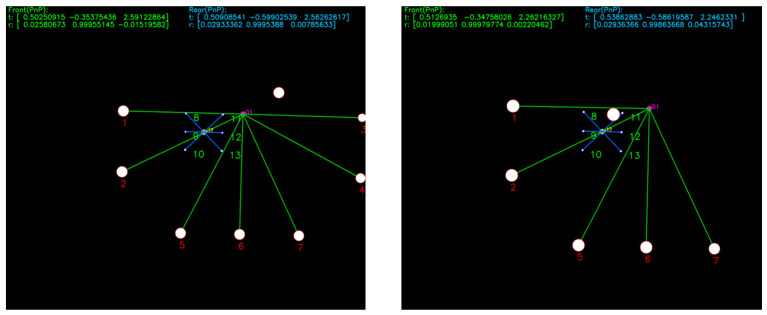
Simulation results under simultaneous spurious and missing beacon conditions.

Furthermore, in continuous guidance tests involving both spurious and missing beacons, conventional one-to-one Hungarian matching may mistakenly associate missing beacons with spurious beacons, resulting in failure of the PSO-based initialization. In contrast, the backtracking Extended Kalman Filter (EKF) leverages the pose history from the previous frame to reliably recover correct beacon tag matching, even under such challenging conditions. Figure 11 illustrates a continuous guidance scenario: in the left image, a spurious beacon appears; in the subsequent right image, a true beacon becomes occluded while the spurious beacon persists. Under this challenging condition, the backtracking Extended Kalman Filter effectively suppresses the spurious beacon and correctly identifies the tags of the remaining real beacons.

(3) Search-Phase Simulation Verification

To further evaluate the algorithm’s adaptability to varying approach directions, the camera was placed 15 m in front of a simulated docking station, with its optical axis perpendicular to the docking plane. It traversed a rectangular path parallel to the docking plane, simulating the docking station entering the field of view from multiple approach directions.

**Figure 12:**
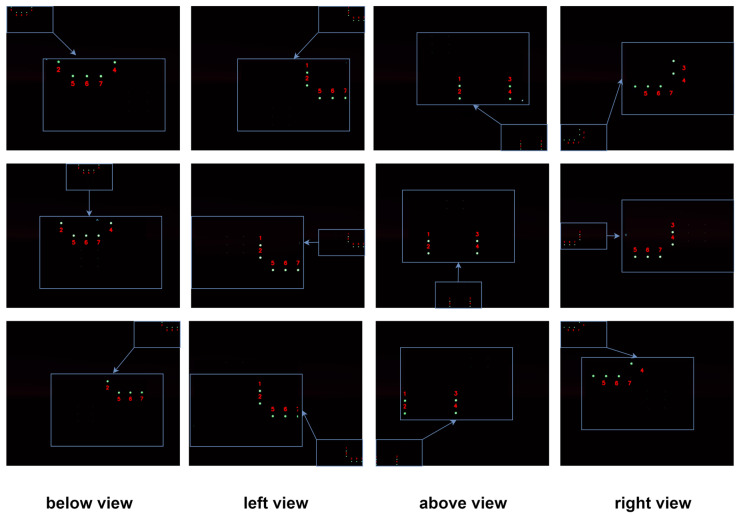
Simulation results in the planar rectangular search experiment.

Figure 12 presents representative matching results when the AUV approaches the docking station from below, left, above, and right. Regardless of the approach direction, as long as at least four lights are visible, the system consistently achieves correct beacon tag matching.

**Figure 13:**
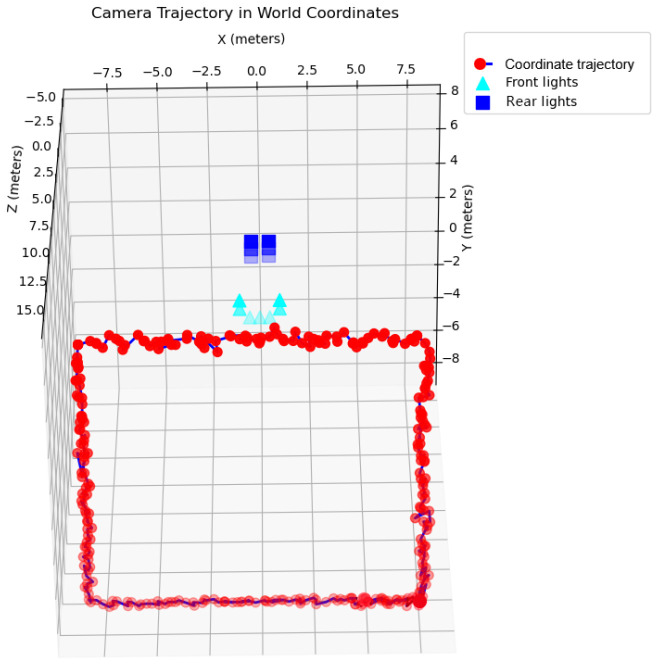
Localization trajectory during planar rectangular path search.

The corresponding camera trajectories in Figure 13 closely match the expected paths, further confirming the algorithm’s robustness and effectiveness under long-distance, multi-directional approach conditions.

### 4.2. Pool-Based Feasibility Experiment for Continuous Guidance

(1) Test Platform and Equipment

**Figure 14:**
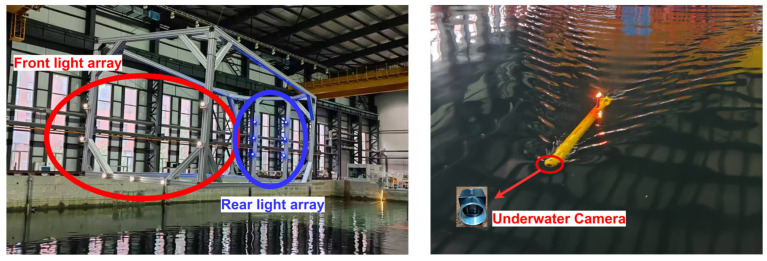
The docking station with dual-layer light array and TS-MINI AUV of SIA.

The experimental setup consists of an LED beacon array, a TS-MINI AUV equipped with an underwater camera (Figure 14), and an onboard processing computer. The TS-MINI, developed by Shenyang Institute of Automation, Chinese Academy of Sciences, has been widely adopted for underwater visual localization research [27,28]. Detailed system specifications are listed in Table 2.

(2) AUV Recovery and Docking Experiment

Prior to the docking tests, the AUV’s camera was calibrated, and the docking rig was suspended in the pool. The AUV initiated its approach from a distance of 30 m at a speed of approximately 1 m/s. Upon detecting the front-layer light beacons, the system started localization based on their observed positions. Once all six rear-layer lights became visible, the system switched to rear-layer guidance. Throughout both the front-layer and rear-layer guidance phases, the AUV was maintained within 1 m of the docking-plane center.

**Figure 15:**
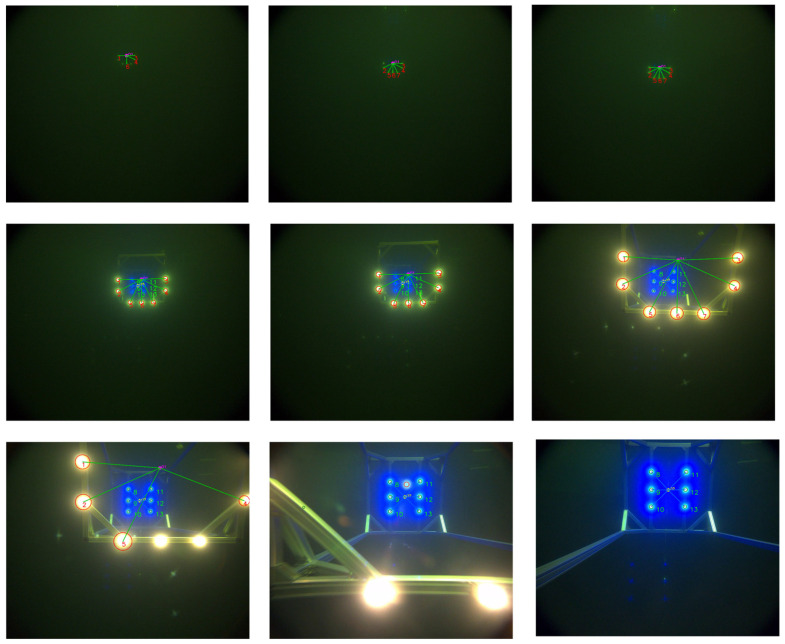
Beacon matching result during the underwater continuous guidance experiment.

Figure 15 shows the tag-matching and center-point localization results produced by the algorithm. Throughout most of the trial, the algorithm reliably identified true beacons, rejected spurious detections, and maintained consistent tag matching. During the early far-distance stage, the beacon signal becomes too weak relative to the background, which reduces the likelihood of successful detection. At very close range, specular reflections from the docking station cause adaptive-threshold segments to merge, interfering with recognition. These effects are expected for optical sensing in underwater environments. Even so, the system can still estimate pose from the remaining visible beacons when some tags are missed. In terms of processing speed, the average detection-and-matching times were 0.112 s for the dual-layer array and 0.097 s for the single-layer array, both below the fixed 0.15 s per-frame interval, fully satisfying real-time constraints.

**Figure 16:**
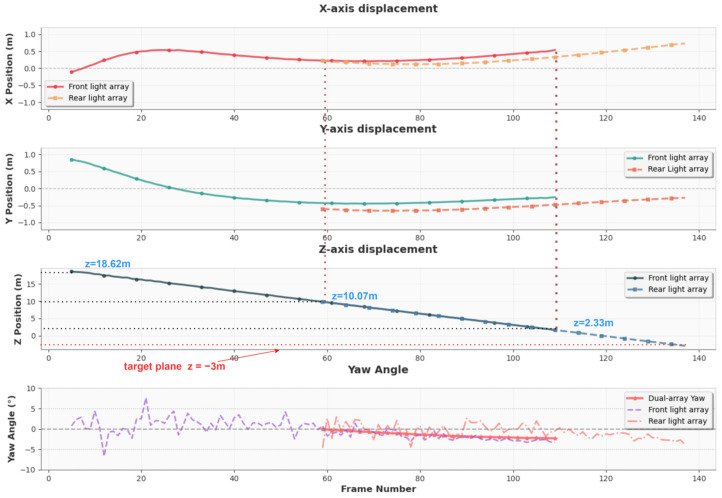
XYZ pose estimation trajectory during continuous guidance.

As shown in Figure 16, the actual localization trajectory is represented by a solid line for the front-layer lights and a dashed line for the rear-layer lights. The experimental data was collected with a fixed sampling interval of 0.15 s per frame. The experimental results show that the localization results of the front-layer lights are available from approximately 18.62 m and terminate at around 1.73 m. The localization results of the rear-layer lights become available from 10.07 m, ensuring continuous guidance coverage throughout the approach sequence. By fusing information from the dual-layer light array, the AUV is guided accurately to the target docking plane at z = −3 m. The localization offset between the center points of the front and rear beacon arrays remained below 0.3 m, which falls within the expected parallax deviation under nominal AUV attitudes, including small pitch, yaw, and roll angles.

## 5. Conclusions

In this work, we present a monocular visual localization method for continuous autonomous docking of underwater vehicles, based on a novel dual-layer guiding light array. To accommodate the varying field during AUV entry into a cage-type docking station, we designed a dual-layer guiding light array and developed a corresponding pose-estimation algorithm. The proposed approach dynamically differentiates front-layer and rear-layer light arrays, overcoming the point-matching challenges inherent in PnP formulations. Whether the dual-layer light array is fully visible or only partially observed, the method consistently enables reliable visual guidance. It effectively addresses partial-visibility pose estimation issues during the search phase, extends the vehicle’s operational search range, and robustly handles both undetected beacons and spurious beacon detections, thereby reducing the risk of localization failure during docking.

Extensive simulations and pool-based experiments verify the robustness of the proposed algorithm. Under extreme conditions with three missing lights or two spurious beacons, it achieves matching success rates of 90.3% and 99.6%, respectively, while the incorporation of a filtering strategy and backtracking extended Kalman filter (BTEKF) raises the success rate to above 99.9%. Regardless of whether the dual-layer light array is fully visible or only partially observed, the method provides consistent and reliable visual guidance throughout the docking process. The system maintains stable, real-time performance with an average detection time of 0.112 s and keeps the localization offset between front and rear beacon arrays below 0.3 m, enabling the AUV to remain within 1 m of the docking-plane center during continuous guidance from a 30 m approach to final docking.

However, certain limitations of the proposed approach should be acknowledged. Although the introduction of geometric consistency correction mechanism and backtracking extended iterative Kalman filter resolves the one-to-one dependency after initialization, the initialization method still relies on Hungarian matching (one-to-one matching). This means that when spurious light sources and missing light sources coexist simultaneously at the beginning, the algorithm may incorrectly match spurious sources to the missing sources, leading to failure in finding correct correspondences. The geometric consistency mechanism cannot obtain correct matching point pairs under such circumstances. This situation often occurs at the initial phase of docking when light source detection is incomplete while spurious light sources caused by interference exist on the water surface, such as reflections. This may result in the inability to initially locate the docking station until all correct light sources are fully detected, potentially shortening the effective localization distance. This limitation needs to be addressed in future work. Furthermore, it should be noted that the proposed method operates downstream of the detection stage, focusing on beacon matching and pose estimation given detected light centroids. A comprehensive study of environmental factors such as water turbidity, salinity, and varying illumination conditions would primarily involve the image acquisition and front-end detection stage, which requires dedicated imaging algorithms and detector-level evaluations. A full imaging and front-end detection study represents an important direction for future work to further enhance the system’s robustness under diverse underwater environmental conditions.

This study provides a valuable technical solution for beacon-based localization in underwater robotic navigation and AUV recovery tasks. Future work will further explore the integration of artificial intelligence technologies to enhance the stability of multi-marker matching and improve adaptability in turbid waters or dynamic marine conditions. Adaptive reward-shaping reinforcement learning methods [29] provide insights for visual guidance deep learning approaches and could be used to optimize the feature extraction and matching strategies in this study through adaptive mechanisms. Recent advancements in swarm intelligence optimization methods [30] could be applied to achieve adaptive parameter tuning of the PSO algorithm in this study through multi-agent mechanisms and knowledge-driven strategies. A hybrid deep learning and geometric optimization end-to-end method [31] offers a potential technical path for constructing the entire visual localization process as an end-to-end trainable network, which is expected to further improve the performance of dual-layer light array matching.

## Figures and Tables

**Table 1:** PSO matching accuracy with backtracking EKF.

Condition	Missing 3	Missing 2	Missing 1	1 Spurious	2 Spurious
PSO	90.3%	98.6%	100%	99.6%	99.6%
PSO + BT EKF	100%	100%	100%	100%	99.9%

**Table 2:** Visual guidance system specifications.

Component	Specification	Quantity
White LED Beacons	Spectrum range: 400–700 nm Power Consumption: 5.4 W Luminous Intensity: 637 cd beam angle: 120°	7
Blue LED Beacons	Wavelength: 455–460 nm Power Consumption: 2 W Luminous Intensity: 127 d	6
	beam angle: 90°	
TS-MINI AUV	Physical dimensions: 160 cm × 10 cm × 10 cm	
Underwater Camera	Sensor Model: Sony IMX264	1
	Effective pixels: 2448 × 2048	
	Field of view: 60°	
	Voltage: 9–24 VDC	
	Pixel size: 3.45 µm × 3.45 µm	
	Frame rate: 15 FPS	
	Focal length: 7.2 mm	
Onboard Computer	NVIDIA Jetson AGX Xavier	1
	CPU: 6-core NVIDIA Carmel ARM	
	GPU: NVIDIA Volta	

## Data Availability

Access to the data will be considered upon request by the authors.
